# Evaluation of changes to foot shape in females 5 years after mastectomy: a case–control study

**DOI:** 10.1007/s10549-017-4183-y

**Published:** 2017-03-07

**Authors:** Iwona Głowacka-Mrotek, Magdalena Sowa, Zygmunt Siedlecki, Tomasz Nowikiewicz, Wojciech Hagner, Wojciech Zegarski

**Affiliations:** 1 Department of Rehabilitation, Collegium Medicum of the Nicolaus Copernicus University in Torun, Marii Curie-Skłodowskiej 9 Street, 85-094 Bydgoszcz, Poland; 2 Department of Surgical Oncology, Oncology Center in Bydgoszcz, Collegium Medicum of the Nicolaus Copernicus University in Torun, Bydgoszcz, Poland; 3 Department of Laser Therapy and Physiotherapy, Collegium Medicum of the Nicolaus Copernicus University in Torun, Toruń, Poland; 4 Department of Neurosurgery, Collegium Medicum of the Nicolaus Copernicus University in Torun, Toruń, Poland

**Keywords:** Mastectomy, Negative consequences, Photogrammetry, Feet

## Abstract

**Purpose:**

The aim of this study was to evaluate changes in foot shape of women 5 years after undergoing breast amputation.

**Methods:**

Evaluation of foot shape was performed using a non-invasive device for computer analysis of the plantar surface of the foot. Obtained results were compared between feet on the healthy breast side (F1) and on the amputated breast side (F2).

**Results:**

128 women aged 63.60 ± 8.83, 5–6 years after breast amputation were enrolled in this case–control study. Weight bearing on the lower extremity on the amputated breast side (F1) compared with the healthy breast side (F2) showed statistically significant differences (*p* < 0.01). Patients put more weight onto the healthy breast side. No statistically significant difference was found with regard to F1 and F2 foot length (*p* = 0.4239), as well as BETA (*p* = 0.4470) and GAMMA (*p* = 0.4566) angles. Highly statistically significant differences were noted with respect to foot width, ALPHA angle, and Sztriter–Godunov index—higher values were observed on the healthy breast side (*p* < 0.001). Highly statistically significant differences were also noted while comparing Clark’s angles, higher values being observed on the operated breast side (*p* < 0.001).

**Conclusions:**

Differences in foot shape on the healthy breast side and amputated breast side constitute a long-term negative consequence of mastectomy, and can be caused by unbalanced weight put on feet on the healthy breast side compared to the amputated breast side.

## Introduction

Breast cancer is one of the most common malignant neoplasms in women. Despite unquestionable advances in the diagnosis and treatment that took place over the past decades, it is still impossible to fully eliminate the risk of side effects of cancer therapy. Treatment of breast cancer, both surgical and adjuvant, can lead to negative consequences, which are usually not associated with the disease itself. Those complications can be distinguished into early—occurring during or immediately after treatment, and late—occurring years after treatment. Early complications include surgical site infection and bleeding, prolonged lymphorrhea, ischemia (necrosis) of wound margins, phantom pain of the amputated breast [1]. Late complications associated with breast cancer treatment include: damage to the long thoracic nerve, skin hyperalgesia of the operated site, scar contracture, muscle contracture, lymphedema [2, 3]. Early as well as late consequences of breast surgery lead to changes in posture. Such changes disrupt the statics of the torso, resulting in increased muscle tension in some muscle groups and contraction in other parts [4–6]. Those factors affect axis of the extremity and foot convexity. Changes to the shape of the foot are important factors leading to loss of mobility and increased risk of falls [7].

The aim of this study was to assess weight distribution and to evaluate foot shape in patients, who had undergone mastectomy for breast cancer 5 years earlier.

## Methods

This was a case–control study conducted with the permission of the Bioethics Committee of Collegium Medicum in Bydgoszcz (No.234/2016) between October 2016 and December 2016 on patients 5–6 years after breast amputation, who were members of the Amazons Clubs in the Kuyavian-Pomeranian Voivodeship. Among 450 members of Kuyavian-Pomeranian Amazons Clubs, 128 women met the inclusion criteria.

Inclusion criteria for the study:

- patients who gave informed consent to participate in the study,
- patients who had undergone unilateral breast amputation at least 5 years prior to the study,
- patients who have not been diagnosed with lymphedema of the upper extremity on the operated side,
- patient age 50–80 years, and
- patients with normal physical function and no difficulties walking.

Exclusion criteria were as follows:

- neurological or musculoskeletal disorders,
- history of trauma causing permanent disruption of normal posture and foot shape,
- upper extremity lymphedema on the operated side. As lymphedema, we defined a difference in circumference of more than 2 cm between the upper extremity on the side of amputated breast and the healthy side in the widest part of metacarpus (excluding the thumb), 10 cm below and 10 cm above the lateral epicondyle of humerus,
- presence of bone metastases,
- patients who had undergone reconstructive surgery,
- psychiatric disorders,
- obesity (3rd degree, BMI > 40),
- other severe conditions (ASA IV), and
- 1st and 4th stage of clinical advancement according to TNM.

The study was conducted according to the following scheme:

- filling out our an information questionnaire (age, employment, place of residence, information regarding adjuvant therapy),
- measuring height and body mass, calculating BMI,
- performing dual-scales test in order to evaluate whether the patient bears similar weight on both feet (patients were wearing external breast prosthesis during this examination), and
- foot shape examination (patients were wearing external breast prosthesis during this examination).

A CQ-ST device by CQ Electronic system was used for foot shape examination. Computer evaluation of the foot is an extension of podoscopic examination, where data on spatial shape of the foot convexity are gathered in addition to footprints. During examination, the patient stands on the podoscope with balanced weight; subsequently, the examiner obtains images of the foot on the screen and is able to analyze specific parameters. In order to assign footprints to a specific type of convexity, obtained results were compared with commonly used norms for adults.

The following parameters were used for foot shape analysis:

- foot length (DL) in mm;
- foot width (SZ) in mm;
- Wejsflog index, i.e., proportion of foot length to food width (DL/SZ), assessing the transverse arch of the foot. The following cut-off points were assumed: high convexity >3°, normal 2.44°–3.0°, and flat <2.44°;
- valgus angle of the hallux (ALPHA angle) in degrees. Reference range ranges: normal 0°–9°, hallux valgus >9°;
- varus angle of the fifth toe (BETA angle) in degrees. Reference range: normal 0°–5°, varus fifth toe >5°;
- heel angle (GAMMA angle) describing transverse arch of the foot in degrees. Reference range: high convexity <15°, normal 15°–18°, flat foot >18°;
- Sztriter–Godunov index (KY) describing the longitudinal arch in degrees. Reference range: pes cavus 0.00°–0.25°, norm 0.26°–0.45°, collapsed arch 0.46°–0.75°;
- Clark’s angle in degrees describing the longitudinal arch of foot. The assumed ranges: pes cavus >55°, norm 42°–54°, fallen arch 20°–41°, flat foot <20°;
- surface of foot touching ground in mm^2^ (PS);
- selected foot shape parameters were compared between the amputated breast side and the healthy breast side in the same individual.

### Statistical methods

Statistical analysis was conducted using PQStat statistical software version 1.6.0.428.

Comparison of foot parameters between the operated side and the healthy side were conducted using paired two-sample Student’s *t* test. This test is used for comparing means considering that both results are obtained from the same person on two different sides.

Comparison of categorized size values on the operated breast side and healthy breast side was conducted using McNemar–Bowker test. This test is used to compare distribution of results considering that both results are obtained from the same person on two different sides.

As statistically significant, we assumed probability level of *p* < 0.05, and as highly statistically significant, we assumed probability level of *p* < 0.01.

## Results

Study group consisted of 128 females that were characterized with respect to their demographic and clinical features. Results are summarized in Table 1. Mean age in the studied group was 63.60, mean weight—71.28 kg, mean height—1.62 m, and mean BMI was 27.35 kg/m^2^. All patients declared participation in physical rehabilitation at least once a week. As much as 20.31% of patients had undergone preoperative chemotherapy, and all patients received adjuvant therapy—usually CHTH + RTH (47.66%). Also, 50.78% of patients declared wearing an external prosthesis during the day, 37.51%—occasionally, while 11.71% would wear it during day and night.

**Table 1:** Clinical characterization and demographics of the studied group

	Arithmetic mean	Median	SD
Age (years)	63.60	65.00	8.83
Weight (kg)	71.28	70.00	11.65
Height (m)	1.62	1.62	0.08
BMI	27.35	26.57	4.97

	Number of patients	Percentage (%)
Operated side		
Right	63	49.22
Left	65	50.78
Place of residence		
Town	60	46.88
Village	68	53.13
Participation in physiotherapy		
1 × per week	32	25.00
2 × per week	43	33.59
3 × per week	53	41.41
Wearing external breast prosthesis		
During day	65	50.78
During day and night	15	11.71
Occasionally	48	37.51
Type of procedure		
Right-sided breast amputation	65	50.79
Left-sided breast amputation	63	49.22
Neoadjuvant treatment		
None	102	79.69
CHTH	26	20.31
Adjuvant treatment		
CHTH	41	32.03
HTH	20	15.63
RTH	6	4.69
CHTH, RTH	61	47.66

*p* statistical significance, *CHTH* chemotherapy, *RTH* radiotherapy, *HTH* hormone therapy

Parameters characterizing foot shape on the operated breast side (F1) and the amputated breast side (F2) were compared. Results are summarized in Table 2. Weight bearing on the amputated breast side (F1) and healthy breast side (F2) showed highly statistically significant difference (*p* < 0.01). Patients put more weight onto the leg on a healthy breast side (F1-AM-34.41; F2-AM-36.82). No statistically significant difference was noted with respect to foot length (*p* = 0.4239), BETA (*p* = 0.4470) and GAMMA (*p* = 0.4566) angles. Highly statistically significant differences were observed with regard to foot width (F1- AM-87.46; F2-91.25), ALPHA angle (F1-9.82; F2-13.05), and Sztriter–Godunov index (F1-0.35; F2-0.46), higher values being reported on the healthy breast side (*p* < 0.001). Highly statistically significant differences were noted with respect to Clark’s angle (F1-52.89; F2-34.18) and PS (F1-75.9; F2-83.74), higher values being observed on the amputated breast side (*p* < 0.001).

**Table 2:** Comparison of foot shape parameters on the amputated breast side (F1) and healthy breast side (F2)

Parameter	F1			F2			Paired two-sample Student’s *t*-test
	Mean	Median	SD	Mean	Median	SD	
Weight bearing	34.41	34.00	6.17	36.82	36.00	7.01	*t* = −3.8707 *p* = 0.0002
Foot length (mm)	230.53	230.00	12.65	230.88	230.00	12.25	*t* = 0.8023 *p* = 0.4239
Foot width (mm)	87.46	88.00	5.88	91.25	91.00	5.26	*t* = −8.5410 *p* < 0.0001
DL/SZ	2.65	2.63	0.20	2.54	2.52	0.15	*t* = −7.7476 *p* < 0.0001
ALPHA angle (°)	9.82	8.05	7.84	13.05	13.00	7.93	*t* = −4.0597 *p* < 0.0001
BETA angle (°)	15.99	16.30	7.46	16.64	15.20	9.09	*t* = −0.7629 *p* = 0.4470
GAMMA angle (°)	15.04	14.80	2.94	15.23	14.90	2.88	*t* = −0.7469 *p* = 0.4566
KY (°)	0.35	0.20	0.24	0.46	0.40	0.27	*t* = −2.9952 *p* = 0.0033
Clark’s angle (°)	52.89	52.50	15.18	34.17	31.30	12.27	*t* = 11.0560 *p* < 0.0001
PS (mm^2^)	75.90	78.00	13.06	83.74	86.80	11.91	*t* = −7.4277 *p* < 0.0001

F1—amputated breast side foot, F2—healthy breast side foot, ALPHA—valgus angle of hallux, BETA—varus angle of fifth toe, GAMMA—heel angle, KY—Sztriter–Godunov index, PS—foot surface touching ground, *p*—statistical significance

Comparison of categorized longitudinal arch values on the amputated breast side (F1) and healthy breast side (F2) showed highly significant (*p* < 0.01) difference with respect to Clark’s angle and significant (*p* < 0.05) difference with respect to KY index. The results are summarized in Table 3. Clark’s angle and KY index indicated pes cavus on the operated side (Clark’s angle F1-48.67%, KY index—51.18%), while on the healthy breast side, they indicated collapse of the longitudinal arch (Clark’s angle F2-71.68%, KY index—37.01%).

**Table 3:** Comparison of categorized value of longitudinal arch of foot on the operated breast side (F1) and healthy breast side (F2)

Longitudinal arch	Clark’s angle				McNemar–Bowker test
	F1		F2		
	Number of patients	Percentage (%)	Number of patients	Percentage (%)	
Pes cavus >55º	55	48.67	10	8.85	*χ* ^2^ = 59.0664 *p* < 0.0001
Normal 42°–54°	36	31.86	16	14.16	
Collapsed arch 20°–41°	20	17.70	81	71.68	
Flat foot <20°	2	1.77	6	5.31	

Longitudinal arch	KY index				McNemar–Bowker test
	F1		F2		
	Number of patients	Percentage (%)	Number of patients	Percentage (%)	
Pes cavus (0.00–0.25)	65	51.18	41	32.28	*χ* ^2^ = 16.2055 *p* = 0.0127
Normal (0.26–0.45)	16	12.60	24	18.90	
Collapsed arch (0.46–0.75)	46	36.22	47	37.01	
Flat foot (0.76–1.00)	0	0.00	15	11.81	

F1—amputated breast side foot, F2—healthy breast side foot, KY—Sztriter–Godunov index, *p*—statistical significance

Comparison of categorized values of transverse arch characteristics on the operated breast side (F1) and healthy breast side (F2) are summarized in Table 4. GAMMA angle analysis did not indicate any statistically significant differences with regard to the feet on the amputated breast side (F1) versus healthy breast side (F2) (*p* = 0.8294). High, normal, and collapse rates for both transverse and longitudinal arches were similar on both sides. Wejsflog index comparison between the amputated breast side and healthy breast side showed statistically significant differences (*p* = 0.0004); normal longitudinal arch was noted in 82.03% of patients on the amputated breast side and in 71.88% on the healthy breast side. High transverse convexity was found in 7 (5.47%) patients on the amputated breast side; collapsed arch was more common on the healthy breast side—36 patients (28.12%).

**Table 4:** Comparison of categorized values of transverse arch characteristics on the operated breast side (F1) and healthy breast side (F2)

Transverse arch	GAMMA angle				Mc Nemar–Bowker test
	F1		F2		
	Number of patients	Percentage (%)	Number of patients	Percentage (%)	
High <15	62	52.10	60	50.42	*χ* ^2^ = 0.8833 *p* = 0.8294
Normal 15–18	42	35.29	43	36.134	
Flat >18	15	12.60	16	13.445	

Longitudinal arch	Wejsflog index				Mc Nemar–Bowker test
	F1		F2		
	Number of patients	Percentage (%)	Number of patients	Percentage (%)	
High >3	7	5.47	0	0.00	*χ* ^2^ = 18.0357 *p* = 0.0004
Normal (2.44–3.00)	105	82.03	92	71.88	
Collapse <2.44	16	12.50	36	28.12	

F1—amputated breast side foot, F2—healthy breast side foot, GAMMA—heel angle, *p*—statistical significance

Table 5 summarizes categorized values of ALPHA and BETA angles.

**Table 5:** Comparison of categorized values of ALPHA and BETA angles on the amputated breast side and healthy breast side

	ALPHA angle				McNemar–Bowker test
	Foot on the operated breast side F1		Foot on the healthy breast side -F2		
	Number	Percentage (%)	Number	Percentage (%)	
0°–9°	67	52.34	27	21.09	*χ* ^2^ = 22.5806 *p* < 0.0001
>9°	61	47.66	101	78.91	

	BETA angle				McNemar–Bowker test
	Foot on the operated breast side		Foot on the healthy breast side		
	Number	Percentage (%)	Number	Percentage (%)	
0°–5°	13	10.16	9	7.03	*χ* ^2^ = 0.45 *p* = 0.5023
>5	115	89.84	119	92.97	

F1—amputated breast side foot, F2—healthy breast side foot, ALPHA—valgus angle of hallux, BETA—varus angle of fifth toe, *p*—statistical significance

Comparison of categorized values of ALPHA angles on the amputated breast side and healthy breast side indicates a highly statistically significant difference (*p* < 0.01). Comparing BETA angles, no statistically significant differences were noted (*p* = 0.5023).

## Discussion

In our study, we evaluated foot shape and weight bearing on lower extremities in patients who had undergone mastectomy due to breast cancer 5–6 years earlier. Devices for computed analysis of the feet were used. It allowed for assessment of longitudinal and transverse arches of the foot, as well as valgus angle of the hallux (ALPHA angle), varus deformity of the fifth toe (BETA angle) and to determine the area of foot surface that touches the ground—PS. Moreover, weight bearing was assessed using a dual-scales test. Our study indicated statistically significant difference in foot shape on the operated breast side (F1) compared to the healthy breast side (F2) with respect to foot width, ALPHA angle, Sztriter–Godunov index, and Clark’s angle (*p* < 0.001). Patients put more weight onto the leg on the healthy breast side (*p* < 0.01).

Longitudinal arch analysis showed that pes cavus was more common on the operated breast side, while pes planus was more common on the healthy breast side. Transverse arch analysis showed that collapse of the transverse arch was more common on the healthy side (F2). ALPHA angle analysis showed that hallux valgus was more common on the healthy breast side.

Current oncologic surgery aims at complete resection with minimal negative consequences. Despite many efforts, multiple negative consequences are observed in breast cancer patients. [1–6] Previous studies indicated susceptibility to developing kyphosis among females after mastectomy [8, 9]. Surgical intervention (together with subsequent adjuvant treatment) causes progression of kyphosis, which results in weakness of muscles of the torso, leading to bone deformities [10].

Studies by other authors indicate that women limit their physical activity after mastectomy, which is the reason for progression of structural deformities of bones and joints. Limitation of physical activity also affects body weight [11]. In our study, mean BMI was 27.35. Studies by other authors emphasize the problem of excess weight in breast cancer patients [12–15]. Bone and joint deformities influence the foot shape [7].

The problem of foot shape is quite commonly encountered in the literature; however, most studies pertain to pediatric patients at different developmental stages [16, 17]. There is a limited number of publications regarding foot shape in adults [7, 18]. Analyzing popular medical databases (Medline, Web of Science), no reports were found concerning the effect of breast amputation on the shape of the foot. Available studies suggest that breast amputation causes changes to bone and joint structure [4–6, 8]. Changes to bones and joints can cause disruption of knee axis and foot deformity [14–17]. Our study suggests a specific tendency with regard to changes in foot shape on the operated and healthy breast side. On the healthy breast side, collapse of the longitudinal arch was more common manifesting as diminished Clark’s angle and increased KY index. On the amputated breast side, there was tendency toward putting less weight on the lower extremity, and elevation of the longitudinal arch could be noted. Analysis of the transverse arch revealed highly statistically significant differences in the Wejsflog index—on the healthy breast side, transverse arch tended to collapse, while on the healthy breast side, it tended to elevate. In some studies by other authors, no differences were observed, while other studies indicated similar differences [18–20]. Our study considered influence of a specific factor on foot shape, and for that reason, no control group was included. Changes to foot shape are associated with age [21, 22]. Changes to foot shape and deformities decrease quality of life. Studies show that they constitute a risk factor for falls and loss of mobility [23–25].

Our study showed that patients did not distribute weight onto both sides equally, and the difference was statistically significant (*p* = 0.0002). There are no similar studies available in the literature. The reason behind unbalanced weight bearing can be due to limited mobility of joints, leg muscle weakness, or abnormal posture.

Adjuvant treatment is an important factor affecting posture among women after mastectomy, causing weakness of postural muscles of the torso and leading to bone deformities [7].

In our study, 58.78% of patients declared wearing external prosthesis during the day and 37.51% occasionally. Not wearing an external breast prosthesis can result in bone deformities and abnormal body axis [26].

In our study, no statistically significant differences were found with regard to the varus angle of the fifth toe (BETA) and heel angle (GAMMA). BETA angle values indicate varus deformity of the fifth toe. It is a characteristic feature in postmenopausal women. It is partly caused by wearing poorly fitting shoes [27–31].

In our study, we observed changes to foot shape despite the fact that all patients declared participation in physiotherapy. In the most commonly applied standard rehabilitation scheme in patients treated for breast cancer, the chief priority is to improve mobility of the shoulder and to protect against or minimize the risk of lymphedema of the upper extremity on the operated side [32, 33].

Mastectomy is an extensive surgical procedure, which can affect patient mobility. In the study by Schultz and Feitis, it was established that motor function could be impaired by such treatment. Patients after mastectomy are characterized by muscle weakness and poor coordination. Disturbances of upper extremity mobility affect lumbosacral part of the spine leading to structural and motor changes and thus improper function of regional muscles, fascia, and ligaments. It can explain progressing deformities of the foot.

Our study has certain limitations despite the fact that tendencies in change of foot shape on the operated breast side and healthy breast side are clear. One of those limitations is lack of initial foot assessment prior to surgery. Therefore, there is a need for similar prospective studies. Such studies would show the dynamics of changes in foot shape. Interesting conclusions could be drawn from information whether such changes relate solely to women treated for breast cancer by breast amputation or also by other surgical methods.

## Conclusions

Our study found that there are differences in foot shape on the operated breast side compared to the healthy breast side in women, who underwent mastectomy for breast cancer,. The reason behind those differences can be unbalanced weight bearing. Our study shows that it is necessary to improve the rehabilitation system for women undergoing mastectomy for breast cancer. Currently, rehabilitation of women after mastectomy is focused on improving mobility of the shoulder girdle on the operated side and reduction of lymphedema. Long-term results and increasing 5-year survival rates show that modern approach to women after mastectomy should also involve gait re-education, working on muscular balance and improvement of static and dynamic balance, for example on a stabilometric platform.

The results of our study on foot shape in women treated for breast cancer indicate the need for broadening the knowledge on this subject and further research on modern methods of rehabilitation.

## References

[1] Kootstra JJ, Hoekstra-Weebers JE, Rietman JS, de Vries J, Baas PC, Geertzen JH, Hoekstra HJ (2010). A longitudinal comparison of arm morbidity in stage I-II breast cancer patients treated with sentinel lymph node biopsy, sentinel lymph node biopsy followed by completion lymph node dissection, or axillary lymph node dissection. Ann Surg Oncol.

[2] DiSipio T, Rye S, Newman B, Sandi H (2013). Incidence of unilateral arm lymphoedema after breast cancer: a systematic review and meta-analysis. Lancet Oncol.

[3] Assis MR, Marx AG, Magna LA, Ferrigno IS (2013). Late morbidity in upper limb function and quality of life in women after breast cancer surgery. Brazil J Phys Ther..

[4] Głowacka I, Nowikiewicz T, Hagner W (2017). Sagittal plane postural changes in female patients with breast cancer after different surgical techniques. Breast J.

[5] Głowacka I, Nowikiewicz T, Siedlecki Z (2016). The assessment of the magnitude of frontal plane postural changes in breast cancer patients after breast-conserving therapy or mastectomy—follow-up results 1 year after the surgical procedure. Pathol Oncol Res.

[6] Ciesla S, Polom K (2010). The effect of immediate breast reconstruction with Becker-25 prosthesis on the preservation of proper body posture in patients after mastectomy. Eur J Surg Oncol.

[7] Moncada LV (2011). Management of falls in older persons: a prescription for prevention. Am Fam Physician.

[8] Bąk M (2008). Body posture in the sagittal plane in post-mastectomy women, who take active part in physical rehabilitation. Physiotherapy.

[9] Szczygieł A, Ciszek E, Materna G (2003). Analysis of selected features of body build and posture in a group of postmenopausal women diagnosed with osteoporosis. Pol J Physiother.

[10] Westrup JL, Lash TL, Thwin SS, Silliman RA (2006). Risk of decline in upper-body function and symptoms among older breast cancer patients. J Gen Intern Med.

[11] Shoff SM, Newcomb PA, Trentham-Dietz A (2000). Early-life physical activity and postmenopausal breast cancer: effect of body size and weight change. Cancer Epidemiol Biomark Prev.

[12] La Vecchia C, Negri E, Franceschi S, Talamini R (1997). Body mass index and post-menopausal breast cancer: an age-specific analysis. Br J Cancer.

[13] Ballard-Barbash R, Schatzkin A, Taylor PR (1990). Association of change in body mass with breast cancer. Cancer Res.

[14] Battaglini CL, Mihalik JP, Bottaro M (2008). Effect of exercise on the caloric intake of breast cancer patients undergoing treatment. Braz J Med Biol Res.

[15] Luctkar-Flude M, Groll D, Woodend K (2009). Fatique and physical activity in older patient with cancer, a six-month follow-up study. Oncol Nurs Forum.

[16] Yang H, Wu J, Cheng J (2017). Is high relative humidity associated with childhood hand, foot, and mouth disease in rural and urban areas?. Public Health.

[17] Cikajlo I, Osrečki K, Burger H (2016). The effects of different types of ankle-foot orthoses on postural responses in individuals with walking impairments. Int J Rehabil Res.

[18] de Vasconcellos Henrique A, de Maia H (2011). The adductor hallucis muscle shapeless and the hallux valgus. Int J Morphol.

[19] Lee DY, Seo SG, Kim EJ (2016). Inter-segmental motions of the foot in healthy adults: gender difference. J Orthop Sci.

[20] Drzał-Grabiec J, Podgórska J, Rykała J (2013). Changes in shape of elderly foot. Postępy Rehabil.

[21] Menz HB, Morris ME (2005). Footwear characteristics and foot problems in older people. Gerontology.

[22] Dawson J, Thorogood M, Marks SA (2002). The prevalence of foot problems in older women: a cause for concern. J Public Health.

[23] Takao M, Komatsu F, Oae K, Miyamoto W (2007). Proximal oblique-domed osteotomy of the first metatarsal for the treatment of hallux valgus associate with flat foot: effect to the correction of the longi-tudinal arch of the foot. Arch Orthop Trauma Surg.

[24] Menz HB, Munteanu SE (2005). Radiographic validation of the Manchester scale for the classification of hallux valgus deformity. Rheumatology.

[25] Nix S, Smith M, Vicenzino B (2010). Prevalence of hallux valgus in the general population: a systematic review and meta-analysis (Review). J Foot Ankle Res.

[26] Glaus SW, Carlson GW (2009). Long-term role of external breast prostheses after total mastectomy. Breast J.

[27] Dawson J, Thorogood M, Marks SA, Juszczak E (2002). The prevalence of foot problems in older women: a cause for concern. J Public Health.

[28] Barnish MS, Barnish J (2016). High-heeled shoes and musculoskeletal injuries: a narrative systematic review. BMJ Open.

[29] Davis AM, Galna B, Murphy AT (2016). Effect of footwear on minimum foot clearance, heel slippage and spatiotemporal measures of gait in older women. Gait Posture.

[30] Trombini-Souza F, Matias AB, Yokota M (2015). Long-term use of minimal footwear on pain, self-reported function, analgesic intake, and joint loading in elderly women with knee osteoarthritis: a randomized controlled trial. Clin Biomech.

[31] de Mettelinge Roman, Calders P, Danneels E, Geeroms S, Du Four C, Cambier D (2015). Does footwear matter when performing spatiotemporal gait analysis among older women?. J Geriatr Phys Ther.

[32] Chan DN, Lui LY, So WK (2010). Effectiveness of exercise programmes on shoulder mobility and lymphoedema after axillary lymph node dissection for breast cancer: systematic review. J Adv Nurs.

[33] Box RC, Reul-Hirche HM, Bullock-Saxton JE, Furnival CM (2002). Shoulder movement after breast cancer surgery: results of a randomised controlled study of postoperative physiotherapy. Breast Cancer Res Treat.

